# Combination of Electroencephalography and Near-Infrared Spectroscopy in Evaluation of Mental Concentration during the Mental Focus Task for Wisconsin Card Sorting Test

**DOI:** 10.1038/s41598-017-00448-6

**Published:** 2017-03-23

**Authors:** Che-Wei Chen, Chia-Wei Sun

**Affiliations:** Biomedical Optical Imaging Lab, Department of Photonics, College of Electrical and Computer Engineering, National Chiao Tung University, Hsinchu, Taiwan

## Abstract

Near-infrared spectroscopy (NIRS) is a noninvasive neuroimaging tool for measuring evoked functional changes in brain oxygenation. Electroencephalography (EEG) can be used to evaluate the functionality of cortical connections and obtain information on regional cortical activity. Coregistration of EEG–NIRS is a recent technique that has been applied for measuring changes in electrical and hemodynamic activity in the human brain. EEG–NIRS coregistration facilitates the avoidance of misleading interpretations of NIRS, particularly in the diagnosis of neurological disorders. In this study, we investigated an approach for enhancing accuracy of NIRS by using EEG to monitor physiological activity during a mental focus task. Using the Wisconsin Card Sorting Test for the subjects mental focus task, we identified two trend types in the EEG and NIRS signals of normal subjects. These data can assist in understanding brain activation statuses and enable determining subjects’ degree of mental concentration. If the data can be standardized for the diagnosis of neurological disorders, they can provide a new index to improve traditional methods (e.g., questionnaires) to assist clinical doctors in diagnosing cognitive disorders.

## Introduction

The Wisconsin Card Sorting Test (WCST) is a common neuropsychological test that has received considerable investigative attention since its introduction in 1948^1–3^. Originally developed to test abstract reasoning abilities and the ability to modify cognitive strategies according to environmental influences in normal adult populations^4^, the WCST was later used to diagnose frontal brain lesions in patients with epilepsy^5, 6^ and to other types of brain lesions^7^, mainly because of its putative sensitivity to frontal lobe dysfunction.

Many studies have applied the WCST to diagnose the damage to the cognitive function of patients with frontal and nonfrontal pathologies. In addition, the WCST has been applied to measure the cognitive performance of healthy older adults, as well as that of patients with major depression, dysthymia, and schizophrenia. To assess the extent of brain activity during the WCST, an instrument must be used for real-time monitoring. Therefore, this study investigated the simultaneous use of electroencephalography (EEG) and near infrared spectroscopy (NIRS). EEG studies have demonstrated the difference between favorable and poor WCST performance by analyzing difference in brain waves frequency^2, 8^. However, studies of NIRS are uncommon, such as those using NIRS during the WCST to determine the change in oxygenation between rest and performing WCST tasks^3^. In the present study, we inferred mental concentration on the basis of changes in oxygenation.

NIRS is one of the most practical and convenient optical diagnostics^9^, because it requires no special equipment or radioactive compound such as radiation exposure. NIRS can measure the changes in oxyhemoglobin (HbO_2_) and deoxyhemoglobin (HHb) concentration in the microvasculature of the brain during time-sequential, real-time monitoring^10^. NIRS is convenient for clinical use because it does not involve medicine or expensive equipment and hence has low initial and operative costs. Although it can be used to obtain a high temporal resolution, a potential disadvantage of NIRS is its shallow photon penetration (3–5 cm); moreover, its spatial resolution is approximately 8 mm, which is inferior to that of functional magnetic resonance imaging.

EEG has long been used for measuring brain function in clinical examinations because of its high temporal resolution. EEG is based on detecting a large population of activated hypersynchronized neurons, mainly localized near the surface of the cortex. However, the EEG method involves placing electrodes on the hair covering the scalp, a requirement that hinders effective low-impedance electrode contact. Consequently, conductive gel is used to reduce the impedance and enable close electrical contact. Because the prefrontal cortex is located in the relatively hairless forehead region, placing electrodes on this area is a feasible option when disease or injury has damaged the other cortices.

For the target analysis techniques, measuring brain function is feasible. Mental conditions can be analyzed by monitoring the cerebral neural activities and dynamic change in blood oxygenation detected by EEG and NIRS. Studies have focused on the frontal cortex, particularly the correlation between frontal cortex activity and mental stress^11–13^.

In the present study, we simultaneously utilized NIRS and EEG to measure prefrontal cortex activity during the WCST understand the brain states involved in mental concentration and active thinking. The two techniques are mutually complementary, because they enable simultaneous analysis of the neuronal and hemodynamic components of brain activity with a high temporal resolution.

## Materials and Methods

### Participants

Thirty adults (18 males and 12 females, mean age = 23.2 ± 4.9 years) were recruited for this study. All subjects were staff or students at National Chiao Tung University. The research was approved by the Institutional Review Board (IRB) of National Chiao Tung University, Taiwan. All methods were performed in accordance with the approval guidelines and regulations. Participation was voluntary, and the participants signed informed consent in accordance with the IRB approval stipulations. The subjects meeting the following inclusion and exclusion criteria were self-selected:

- Participants must not have any metabolic, cardiovascular, respiratory, psychiatric, or drug- or alcohol-related conditions that could affect the measurements or their ability to adhere to the experiment protocol.
- Participants must have normal, or corrected-to-normal vision.
- Participants must have no prior experience with the WCST.

If a participant felt sick at any time, the experimental task was immediately terminated.

### Materials and data acquisition

#### NIRS recording and analysis

The hemodynamic responses were measured with a PortaLite (Artinis, Netherlands), which emits near-infrared rays in two wavelengths (750 and 850 nm) toward the brain through optical fibers attached on the scalp. The instrument features three pairs of intensity-modulated laser diodes (each pair emits two wavelengths, 750 and 850 nm) and one gain-modulated photomultiplier tube detector that simultaneously applies the modified Beer–Lambert law and spatially resolved spectroscopy methods to measure the concentration of HbO_2_ (μM ∙ min^−1^) and HHb (μM ∙ min^−1^) in the region of interest. The distances between the three pairs of sources and detector are 30, 35, and 40 mm, respectively. In this experiment, the probe was affixed to the middle of the frontal cortex region by using a sports band to exert light, comfortable pressure for effective contact between the probe and the subjects’ skin. All data were obtained by using the Oxysoft program (Artinis, Netherlands) with the sample rate set at 1 Hz. The data were then normalized before additional calculations were performed to provide a common scale for the variables. Subsequently, the mean and standard deviation were obtained for the concentration of HbO_2_ and HHb.

#### EEG recording and analysis

EEG signals were recorded with the PowerLab 15 T (ADInstruments Pty Ltd., Australia), which has five tin electrodes. In accordance with the International 10–20 system, we placed two electrodes at the left frontal (FP1) and right frontal (FP2) positions, two reference electrodes at the locations A1 and A2 (the earlobes), and the final electrode at A2 to be the ground electrode. The sampling rate was 1 kHz. We used the LabChart 6.1 program (ADInstruments Pty Ltd., Australia) for the analogue-to-digital conversion and storage. The EEG data were filtered using a band-pass of 0.1–40.0 Hz and processed by fast Fourier transform in MATLAB (R2014a). We analyzed the difference between the left frontal and right frontal signals.

#### Stimulus and task

The WCST was used to activate the frontal lobes, inducing the changes in blood oxygenation. The WCST consists of a maximum of 128 cards (i.e., 64 cards repeated once) and a minimum of 60 cards when the three categories (i.e., color, shape, and number) are completed without mistakes. According to the WCST manual^14^, each trial begins with the onset of a compound stimulus comprising four WCST reference cards located below a response card that is centered in the middle of a computer monitor. Participants were requested to choose one in four reference cards to match the response card according to one of three categories (i.e., color, shape, and number). They received the feedback on the display after making each choice, and considered the feedback to make the next choice. One category was completed when ten correct reference cards were matched consecutively. The category was then changed without notice and participants had to identify the new rule correctly. When the participants successfully identified three categories twice, the test was completed. During the baseline and task measurements, data were obtained by the EEG and NIRS systems and stored on a hard disk.

#### Procedure

The experiment was performed in a sound-attenuated room with air conditioning. Participants were seated on a comfortable chair and encouraged to relax. To minimize error, the participants were instructed to remain still while the measurements were performed. A computerized version of the WCST was displayed at a distance of 100 cm at eye level. The mental tasks used in the experiment were as follows:

- Initial rest: Participants closed their eyes and cleared their minds (2 min).
- WCST task: Participants performed the WCST.
- Final rest: Participants closed their eyes and relaxed (2 min).

While the participants performed these tasks, the NIRS and EEG systems recorded the hemodynamic and electrical signals. For the convenience of the participants, the entire experiment was performed in a 30 min period, which included 10 min of preparation time.

## Results

**Figure 1:**
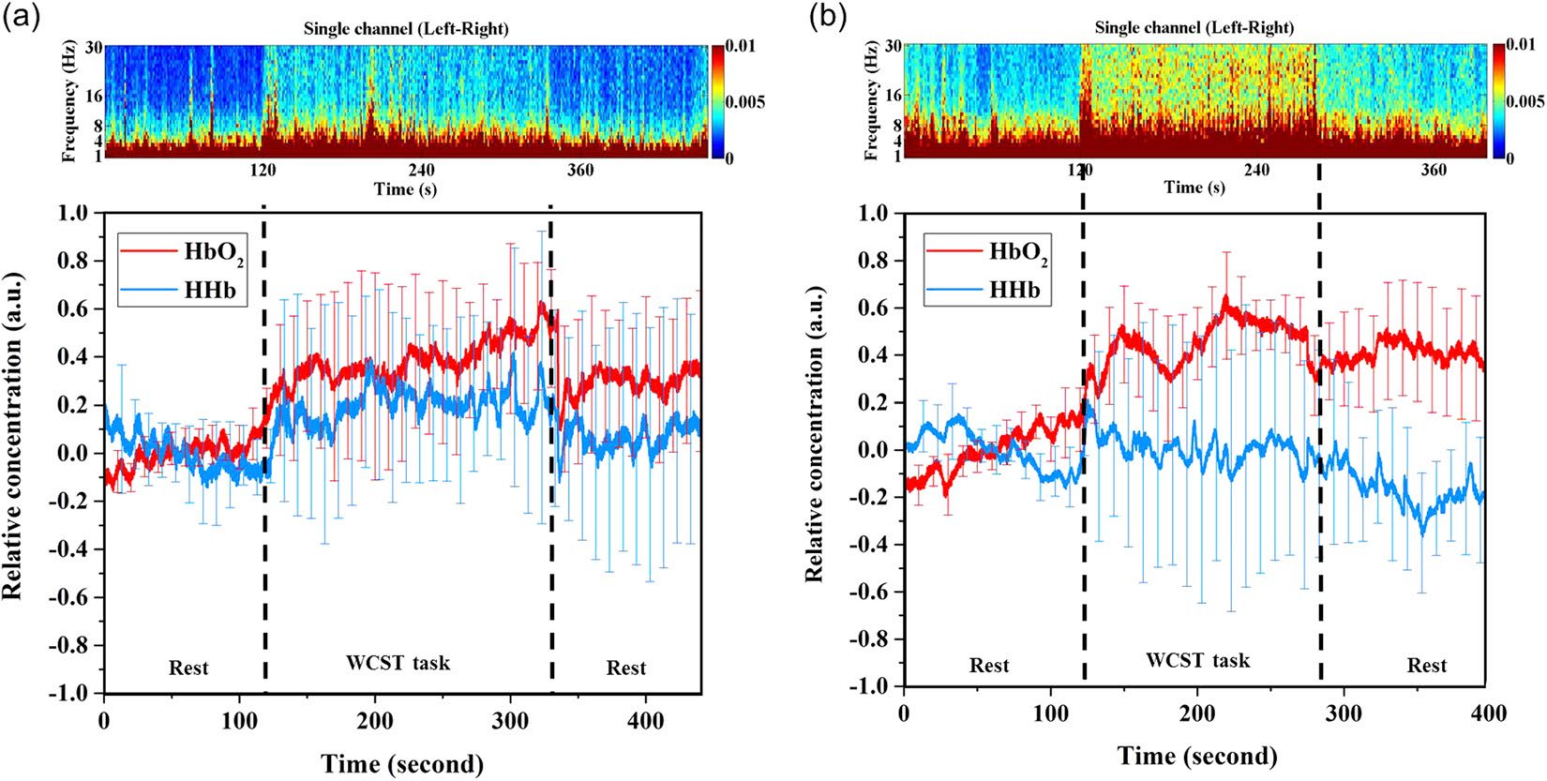
NIRS and EEG data for (**a**) Group 1 and (**b**) Group 2. The top portions of the figure depict the difference between the left channel (FP1) and right channel (FP2) EEG signals. The bottom portions of the figure show NIRS signals for the blood oxygenation changes. The red line is the HbO_2_ concentration and the blue line is the HHb concentration. The x-axis is the experimental time and the y-axis is the change in concentration.

Thirty participants were classified into two groups depending on the number of categories they completed; this reflected their abstract thinking^15, 16^ and problem solving^15^ abilities. Table 1 shows some of the parameters obtained from the WCST task. We used these parameters to analyze the difference between the two groups. For Group 1, the average number of categories completed was 3.7. Each of the 17 participants used up all of the card. Participants in this group exhibited a slow rise in NIRS intensity during the rest and WCST task periods and exhibited low amplitude in the high frequency EEG patterns (Fig. 1). In Group 2, the 13 participants completed all six categories and exhibited substantial differences in blood oxygenation changes during the WCST task according to the NIRS signal. In this group, the high frequency electrical signals differed considerably (Fig. 1).

**Table 1:** WCST parameter with values averaged for each group.

	Group 1	Group 2
**Number of categories completed (a.u.)**	**3.7** ± **1.3**	**6**
Experimental time (sec.)	603.4 ± 97.6	420.2 ± 31.7
Total number of trials (a.u.)	128	89.7 ± 15.1
**Total number of correct (a.u.)**	**66.4** ± **11.2**	**70** ± **8.2**
**Total number of errors (a.u.)**	**61.5** ± **11.2**	**19.7** ± **7.9**
Perseverative responses (a.u.)	13.9 ± 4.8	6.4 ± 2.5
**Percent perseverative responses (%)**	**10.9** ± **3.8**	**7.1** ± **2.7**
Failure to maintain set (a.u.)	1.1 ± 0.8	0.5 ± 0.84
**Trials to complete first category (a.u.)**	**23.4** ± **9.6**	**14.5** ± **4.5**

Figure 1 shows the total average EEG and NIRS intensities for the 30 participants. The top portions depict the EEG signals, which represent the difference between the left channel (FP1) and right channel (FP2). The x-axis is the experimental time and the y-axis is the signal frequency. The color bar represents the intensity range from 0 to 0.01 (a.u.). Most cerebral signals observed through the scalp EEGs fall within the 1–30 Hz range. The waveforms are subdivided into bandwidths known as delta wave (0–4 Hz), theta wave (5–8 Hz), alpha wave (9–16 Hz), and beta wave (17–30 Hz)^17, 18^. The bottom portion of Fig. 1 shows the blood oxygenation changes according to the NIRS signal; the red line is the HbO_2_ concentration, the blue line is the HHb concentration, and the error bar is an indicator line that spans a range. The x-axis is the experimental time and the y-axis is the change in concentration.

In Group 1, the HbO_2_ concentration gradually increased during the initial rest period (0–120s), and the frontal cortex electrical signals comprised only delta and theta waves. During the WCST task (120–335s), the HbO_2_ concentration increased sharply and the electrical signal intensity of the alpha wave grew, whereas that of the beta wave increased only slightly. Throughout the final rest period (335–455s) the HbO_2_ concentration increased gradually. HHb levels did not differ substantially during the entire experiment. However, the electrical signals became weaker than those obtained during the WCST task.

In Group 2, the HbO_2_ level did not differ considerably during the initial rest period (0–120s), and the electrical signals of the delta and theta waves were stronger relative to those of Group 1. During the WCST task (120–275s), the HbO_2_ level differed substantially from that during the initial rest period, and the alpha and beta waves were more intense. During the final rest period (275–395s) the HbO_2_ level was lower than that during the WCST task, and the alpha and beta waves signals were also weaker.

## Conclusion

In the present study, we observed that the trend of the HbO_2_ concentration was consistent with that of that electrical signals during the WCST task. This phenomenon may be determined by how intensely the participants concentrated on the WCST task. According to Table 1, Group 2 had a shorter total experiment time than did Group 1 and necessitated fewer trials to successfully complete the first category and select correct cards^19^. The participants in Group 2 ascertained the rules of the computerized WCST quicker than did the participants in Group 1. Hence, we can conclude that the degree of mental concentration of the participants were better in Group 2 than in Group 1. Nowadays, the degree of mental concentration is always assessed by questionnaires in the clinic, therefore we utilize WCST results as a preliminary discriminant criterion in this study.

**Figure 2:**
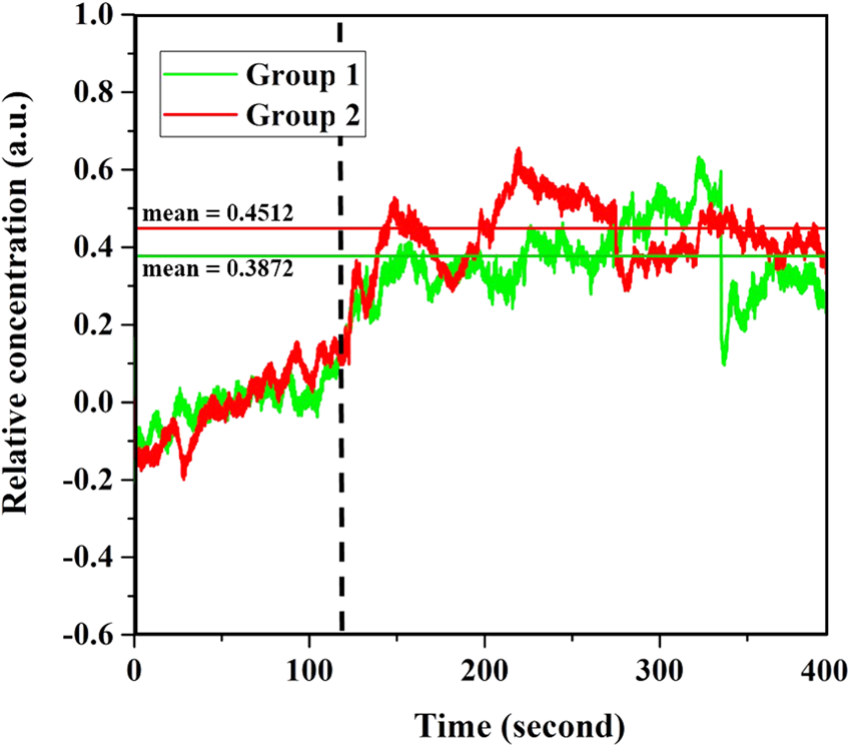
Oxyhemoglobin concentrations of Groups 1 and 2. The green line is Group 1. The red line is Group 2.

Figure 2 depicts the trend in HbO_2_ concentrations for Groups 1 and Group 2 and indicates that the HbO_2_ concentration peaked more quickly for Group 2 during the WCST task (Fig. 2). Furthermore, the mean HbO_2_ concentration for Group 2 (mean = 0.4512) was higher than that for Group 1 (mean = 0.3872) during the WCST task, indicating the HbO_2_ concentration kept higher level for Group 2 subjected in the WCST task. On the basis of the trends and averages, we can infer that the degree of concentration of the participants in Group 2 was better than that of the participants in Group 1. Indeed, the NIRS signals agreed with the discriminant criterion from the WCST results.

## Discussion

EEG has long been used for recording neuronal activities of brain in clinical examinations. EEG measures the electrical signals originated directly from the active neurons, and NIRS measures the change of oxygenation resulting from metabolism of the active neurons. Both of EEG and NIRS signals have the correlation with functional change of neurons. According to the experimental results, the HbO_2_ levels detected by NIRS were consistent with the EEG signals during the WCST task. The HbO_2_ levels and the alpha and beta waves intensities were weaker in the Group 1 participants than in Group 2 participants (Fig. 1). The dynamics of the HbO_2_ levels indicated the brain states entailed in mental concentration and active thinking. The EEG signals confirmed the reliability of NIRS. Furthermore, the EEG signals distinguished the beginning of the WCST task more clearly. EEG and NIRS signals both reflected the extent of brain activity in real-time. Thus, the combination of EEG and NIRS help us can facilitate research into brain activation states. Multimodality imaging techniques will play an important role in clinical examination. Though there were not clear results about the technique merit of combining EEG and NIRS in this study, we proved that NIRS signals can reflect the activation states of brain neurons and have a correlation with EEG signals. Hence, we utilized these real-time blood oxygenation and electrical signals to determine the cognitive state of the participants. However, factors such as intelligence quotient and response capacity must be considered. In the further study, we will continue the research work of coregistration of EEG–NIRS system ﻿for clinical diagnosis in neurology and psychiatry. The new quantitative﻿ indicator b﻿ased on the measured data﻿ will be ﻿found to im﻿prove the traditional psychological ﻿tests for precision medicine of cognitive disorders.
